# Examining the diffusion of coronavirus disease 2019 cases in a metropolis: a space syntax approach

**DOI:** 10.1186/s12942-021-00270-4

**Published:** 2021-04-29

**Authors:** Yepeng Yao, Wenzhong Shi, Anshu Zhang, Zhewei Liu, Shuli Luo

**Affiliations:** 1 Department of Land Surveying and Geo-Informatics, Smart Cities Research Institute, The Hong Kong Polytechnic University, Hong Kong, People’s Republic of China; 2 Department of Geography and Resource Management, The Chinese University of Hong Kong, Hong Kong, People’s Republic of China

**Keywords:** COVID-19, Built environment, Space syntax, Geographically weighted regression

## Abstract

**Background:**

The urban built environment (BE) has been globally acknowledged as one of the main factors that affects the spread of infectious disease. However, the effect of the street network on coronavirus disease 2019 (COVID-19) incidence has been insufficiently studied. Severe acute respiratory syndrome coronavirus 2, which causes COVID-19, is far more transmissible than previous respiratory viruses, such as severe acute respiratory syndrome coronavirus, which highlights the role of the spatial configuration of street network in COVID-19 spread, as it is where humans have contact with each other, especially in high-density areas. To fill this research gap, this study utilized space syntax theory and investigated the effect of the urban BE on the spatial diffusion of COVID-19 cases in Hong Kong.

**Method:**

This study collected a comprehensive dataset including a total of 3815 confirmed cases and corresponding locations from January 18 to October 5, 2020. Based on the space syntax theory, six space syntax measures were selected as quantitative indicators for the urban BE. A linear regression model and Geographically Weighted Regression model were then applied to explore the underlying relationships between COVID-19 cases and the urban BE. In addition, we have further improved the performance of GWR model considering the spatial heterogeneity and scale effects by adopting an adaptive bandwidth.

**Result:**

Our results indicated a strong correlation between the geographical distribution of COVID-19 cases and the urban BE. Areas with higher integration (a measure of the cognitive complexity required for a pedestrians to reach a street) and betweenness centrality values (a measure of spatial network accessibility) tend to have more confirmed cases. Further, the Geographically Weighted Regression model with adaptive bandwidth achieved the best performance in predicting the spread of COVID-19 cases.

**Conclusion:**

In this study, we revealed a strong positive relationship between the spatial configuration of street network and the spread of COVID-19 cases. The topology, network accessibility, and centrality of an urban area were proven to be effective for use in predicting the spread of COVID-19. The findings of this study also shed light on the underlying mechanism of the spread of COVID-19, which shows significant spatial variation and scale effects. This study contributed to current literature investigating the spread of COVID-19 cases in a local scale from the space syntax perspective, which may be beneficial for epidemic and pandemic prevention.

**Supplementary Information:**

The online version contains supplementary material available at 10.1186/s12942-021-00270-4.

## Introduction

The coronavirus disease 2019 (COVID-19) pandemic is one of the most severe global infectious disease pandemics in human history. Severe acute respiratory syndrome coronavirus 2 (SARS-CoV-2), which is the cause of COVID-19, can be transmitted by respiratory droplets or body contact. COVID-19 has thus become a city-level airborne epidemic that will continuously threaten human health [42]. Governments, research institutions, and companies are attempting to defer the propagation of this disease.

In the early to mid-stage of the COVID-19 pandemic, most spatial analyses of the development of COVID-19 have been at the national and provincial levels [40, 49]. With the continuous development of the epidemic and the increase of cases around the world, there emerge a vast body of research that exploring the spread of COVID-19 on city scale or even more microscopic community scale [13, 19]. These research emphasize on various perspectives, including the space–time pattern of COVID-19, human mobility and the spread of disease, the onset risk and the BE. Among these previous research, a large number of studies have analyzed the BE by considering various aspects such as building density, city infrastructure, public facilities and services to study the spreading mechanism of the COVID-19 epidemic.

Street network, an essential component of urban BE, is critical in analyzing the distribution pattern of disease which could spread easily via human interactions on the urban road network. Accordingly, many studies have highlighted the importance of the street network in the spread of infectious diseases [31, 46]. While traditional research usually operationalizes street network as measures of intersection density, the spatial configuration of street network and how it contributes to COVID-19 cases distribution at the city level has not been thoroughly studied [39]. That is, a more integrated street would attract more human activities and thus a higher chance of infection.

To fill this knowledge gap, this study aimed to envisage how and to what extent the COVID-19 is disseminated on a city scale concerning the spatial configurations of urban environment in Hong Kong, a densely populated and highly developed metropolis. Six space syntax and topological measures of the spatial configuration of road networks, along with one confounding variable population density, were introduced to model the relationship. In addition, several regression models including ordinary least squares (OLS), classic geographically weighted regression (GWR) model, and an advanced GWR model with adaptive bandwidth were applied to model the relationship between these measures and the spatial distribution of COVID-19 cases in the past 10 months. The spatial configuration based research framework is generalizable to other cities or regions with high density and urban complexity. Our conclusions’ universality can be verified and perfected by integrating epidemic data and online freely accessible spatial data of other areas.

The remainder of this paper is organized as follows. “Related work” section reviews the previous related work. “Materials and methods” section introduces the study area, the basic theory of space syntax, and our methodology. “Experiment results” section presents the results of our case study in Hong Kong, including the descriptive statistics of initial results and the further spatial regression analysis. “Discussion and conclusion” section summarizes and discusses the study.

## Related work

### Geographical patterns of COVID-19 on different scale

Nearly 1 year since the beginning of the pandemic, numerous studies of COVID-19 have been published that focus on the spread of the disease at the country/district level [3, 8, 40, 49]. Ye and Hu [47] indicated that the emergency-response control measures that were implemented in January 2020 in the Yangtze River Delta region in China were effective, based on their tracking of the spatial and temporal distributions of COVID-19 cases in this area. Mollalo et al. [29] explored the county-level variations in COVID-19 incidence across the continental United States, and found that factors such as income, percentage of black females, and percentage of nurse practitioners significantly contributed to spatial variation in disease incidence. Lakhani [24] identified a priority area in Melbourne, Australia, in which a high percentage of aging adults resided but lacked access to necessary healthcare facilities. This study helped policymakers to allocate related resources and pandemic palliative-care services.

### Effects of the urban road network on disease spread

Although spatiotemporal patterns of COVID-19 cases have been well documented on both macro-scale and micro-scale from a traditional urban BE perspective, limited research has focused on patterns at the local-level urban road network configuration. The urban BE constitutes the bulk of human-made spaces and objects, including buildings, roads, transit networks, and other public areas [12]. As the urban BE is the main space in which humans move and interact with each other, it increases the risk of exposure to pathogens through close contact with the people and surfaces in their daily lives. Poor urban and building design can harm health by increasing the risk of exposure to infection [37]. As one essential component of urban BE, the urban road network configuration has a strong advantage in explaining the human activities across the urban area. As the urban road network is the primary potential area for the transmission of pathogens, due the movement and interaction of large numbers of people. The study of the correlation between the urban road network and pandemics has been part of the history of urbanization since John Snow drew a cholera map of London in 1854, which is recognized as the earliest known example of using geographical inquiry to understand a health epidemic [28]. The literature has suggested the importance of the road network in the spread of infectious diseases. Strano et al. [41] suggested that it was valuable to understand how regions are connected through road network After studying the spread of malaria in Africa. By studying the travel patterns and epidemic, some scholars find that imposing restrictions on specific nodes and in the network can inhibit the spread of infectious diseases [31]. After studying the distribution of more than 40,000 dengue fever cases, Li et al. [25] found that narrow and high-density urban road network greatly contribute to the dengue fever epidemic in Guangzhou and Foshan, China. Similarly, Xu et al. [46] revealed that the centrality of highway networks had a significantly positive relationship with the incidence of swine flu (H1N1 influenza A) in 2009 at the city level in mainland China.

### Space syntax and the urban BE

Despite a burgeoning concern of classically built environment components such as density and land use mix to examine the disease spread, some argue that those measures fail to evaluate the spatial configuration of a street network, which is highly associated with human movement [10, 20, 22]. Although the density is typically used to represent the street layout as the number of intersections within an area, street configuration highlights relationships between streets within the network. For instance, more integrated streets are more likely to attract more human interactions as they are more accessible from other streets. In this regard, space syntax has been widely adopted as a representation of urban space to characterize the spatial configuration of the built environment in relation to human activities (e.g., [17, 36]).

The space syntax theory, proposed by Bill Hillier and colleagues in the 1970s, posits that spatial configuration can explain a large proportion of various human activities, such as pedestrian movements through streets, land-use patterns, and public health [11, 32]. Methodologically, space syntax abstracts real urban space from a continuous entity into a group of axial lines or subspaces, which resolves the complexity of urban areas. Space syntax is based on traditional graph theory and network analysis, and a series of specific space syntax measures have been proposed to indicate or describe urban spatial layouts. These measures showed good fitness in the past decades for investigating the through-movement potential of each street or space in a given area in terms of pairs of other areas. Previous studies have also suggested that space syntax is effective at depicting human activities by decomposing space into line segments and region in urban areas. For instance, Xia et al. [45] proposed an urban growth boundary model based on the space syntax theory, which predicts urban boundary expansion. The model performed better than another that did not incorporate space syntax predictors, and thus suggested that space syntax has great potential for use in urban planning applications. Koohsari et al. [21] applied the space syntax theory to connect urban form and urban function to pedestrian movement, and concluded that space syntax has great potential for use in public health studies.

Spatial variables like road density and building density represent the urban space have long been used to investigate the spatial pattern of infectious diseases in the history of health geography. However, most COVID-19 related researches emphasize socioeconomic perspective and generalized BE such as demographic income, availability of public facilities, to explain how the disease transmits and distributes within a city or state on the fixed scale. This study aims to explore if and how the spatial configuration correlated with the COVID-19 disease on the variable scale within a city and contributes to the epidemic prevention and control strategies.

## Materials and methods

### Study area

**Fig. 1:**
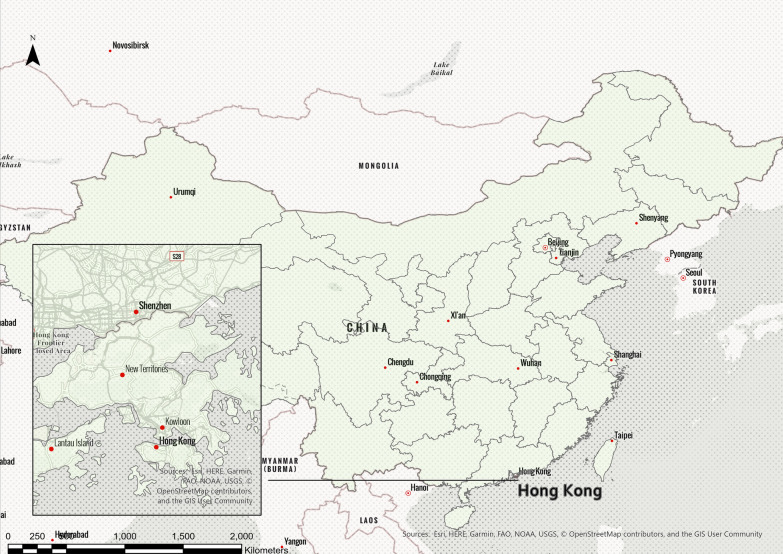
Geographical location of Hong Kong

Hong Kong, with an average population density of 6754 persons per square kilometer, is one of the most densely populated and highly developed metropolises in the world (Fig. 1). Its urban BE has positively contributed to the spread of various infectious diseases. For example, Hong Kong was the most severely affected area of the world during the SARS epidemic in 2003, as it had more than 1700 confirmed cases and 299 deaths. More than 300 cases were distributed in a few blocks of a large high-rise housing estate located in the densely populated district of Kowloon [48]. Epidemiologists found that the high chance of social contact in high-density Hong Kong could have been a major contributor to the high rate of SARS infection. During the COVID-19 pandemic in 2020, the most common control measures in Hong Kong have been self-quarantining and physical distancing. However, strict physical distancing is very difficult to maintain in overcrowded urban spaces, thereby leading to an increased risk of the spread of COVID-19 [1]. Consequently, local cases increased significantly during the second and third waves of the pandemic (early March to early April and late June to late October 2020) while a large number of imported cases comprised the first wave (January to March 2020). Particularly, by the end of the October 2020, the third wave had brought more than 4000 new cases and lead to a severe epidemic in Hong Kong.

### Data collection and processing

**Fig. 2:**
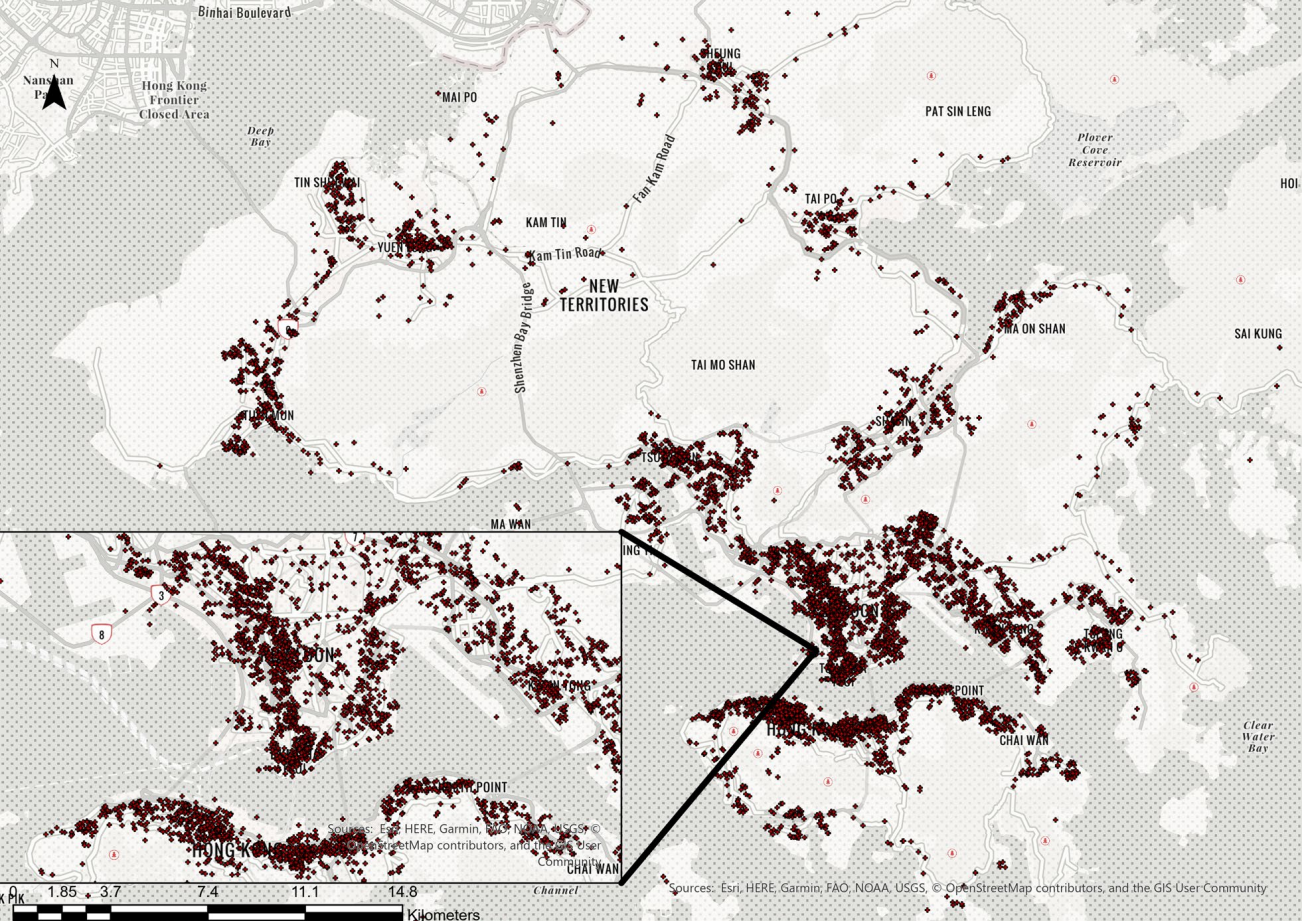
Geographical distribution of cases in the study area

COVID-19 incidence data were collected from the geospatial COVID-19 dashboard (co-designed by the Center of Health Protection, Department of Health, Development Bureau, Lands Department, and a group of volunteers in Hong Kong) for January 18 to October 5, 2020. The dashboard provides details of all of the confirmed cases (a confirmed case is an individual who had a confirmatory viral test performed by way of a throat swab, nose swab or saliva test and that specimen tested positive for SARS-CoV-2, which is the virus that causes COVID-19), including the demographics of each case, a brief travel history, and the locations recorded during the incubation period. We extracted the geographical locations of those cases at the time of their public announcement on the dashboard. After excluding 1299 imported cases and cases in Quarantine Centers, a total of 3815 confirmed cases were extracted for this research (Fig. 2). The study area was divided into a grid of cells that have a resolution of 1 km × 1 km. In order to focus the research on the areas with more daily activities, the non-built-up areas such as country parks, wasteland, and idle sites are excluded based on the official data released by the Hong Kong Lands Department.

### Space syntax measures

**Fig. 3:**
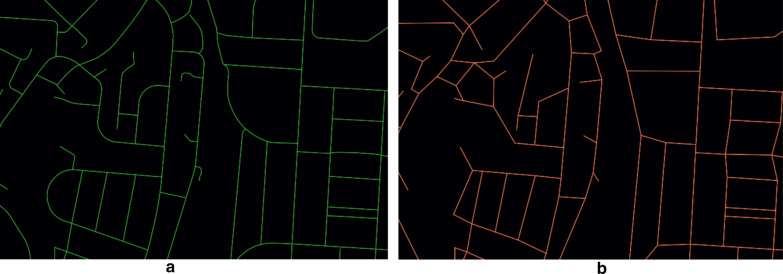
**a** Road-centerline map and **b** axial graph of a locality in the study area

In this study, the urban BE was quantitatively depicted with several space syntax measures. Space syntax seeks to represent urban space as axial lines, and thus differs from traditional segment-node-based network measurements. Specifically, an axial graph represents urban space based on two principles, namely that (1) an axial line representing a particular space must be the longest line in the space and (2) the number of lines must be the lowest possible. Figure 3 illustrates the conversion of a road street map to an axial graph, in which many stubs and nodes are removed.

After the axial graph of the study area was constructed, six classic space syntax measures, namely *degree, control value*, *mean depth, local depth, integration,* and *betweenness centrality*, were computed, using data from the dataset, as the indicators for the urban BE*.* The widely used geometric indicator *length* was also used.

Specifically:

- *degree* (Eq. 1) is a local measure that specifies the number of segments intersecting with the given segment.
- *depth* (Eq. 1), comprising *local depth* and *global depth*, is calculated by counting the steps from other segments to the current segment, as follows:
  $$degree = n_{1}$$
  1 $$depth = \mathop \sum \limits_{k = 1}^{s} n_{k} , \left\{ {\begin{array}{*{20}c} {l > s > 1 \left( {local\;depth} \right)} \\ {l = s \left( {global\;depth} \right)} \\ \end{array} } \right.$$
  where *s* is the number of steps of the given segment, *n*_*k*_ is the number of intersected segments (neighbors) at step *s*, and *l* is the network diameter.
- *control* (Eq. 2) is a measure that describes how a segment controls access to its neighbors from other segments and is calculated by summing the inverse values of the connectivity of all of the segments neighboring a specific segment.
  2 $$Control = \mathop \sum \limits_{k = 0}^{m} 1/n_{k}$$
  where *m* is the number of neighbors and *n*_*k*_ is the number of neighbors of the current neighbor.
- *Integration* (Eq. 3) is a measure that evaluates the distance from a starting point to all points in a given system. A positive correlation was found between the integration of a space and the chance of people’s appearance in that space [9].
  3 $$\left\{ {\begin{array}{*{20}c} {Integ_{i} = \frac{{2\left( {MD_{i} - 1} \right)}}{{\left( {n - 2} \right)}} } \\ {MD_{i} = Global\;depth_{i} / \left( {n - 1} \right) } \\ \end{array} } \right.$$
  where $MD_{i}$ is the mean depth, $Integ_{i}$ is the integration, and *n* is the total number of nodes in the network.
- *Betweenness* (Eq. 4) is a critical measure of spatial network accessibility, which shows how local space controls and mediates the movement and connections through an entire network.
  4 $${\text{B}}\left( x \right) = \mathop \sum \limits_{x \ne y \ne z} \frac{{\delta_{yz} \left( x \right)}}{{\delta_{yz} }}$$
  where *x*, *y*, and *z* are three different nodes in the network and $\delta_{yz}$ represents for the number of shortest paths from *y* to *z*. $\delta_{yz} \left( x \right)$ is the number of times node *x* falls on the shortest path of *y* to *z*. The betweenness centrality is considered at a given radius of 1200 m, which is an approximately 15-min walk for an adult [44].

### Global models and local models

Two regression methods were used to examine how space syntax measures can explain the distribution of COVID-19 cases in Hong Kong, namely a linear regression model based on OLS and a local spatial model based on GWR. In both regression models, the dependent variable is the number of COVID-19 cases in each 1-km grid cell. The independent variables are the values of the space syntax measures with one confounding variable, the population density in each cell.

As discussed in “Related work” section, we conducted both univariate and multivariate regressions to examine the relationships between the selected explanatory variables and the dependent variable. The multivariate regression models were developed globally and locally to determine the most explanatory model for our study area. The OLS approach investigated the relationship between COVID-19 cases and the urban BE across the study area. The GWR model with a fixed bandwidth was applied to examine the OLS results. Finally, we introduced an adaptive bandwidth to calibrate the GWR model results, which enabled the spatial regression model to explain most areas at a local scale.

#### Linear regression based on OLS

The OLS-based linear regression calculates the relationship between the number of COVID-19 cases and the space syntax measures using two basic assumptions, namely that (1) the error terms are independent and have a constant variance across the study area and that (2) the independent variables are not correlated with the error terms [29]. For the model with six variables, the equation used is as follows (Eq. 5):

5 $$y = \beta_{0} + \mathop \sum \limits_{i = 1 \ldots 6} \beta_{i} x_{i} + \varepsilon$$

where *y* is the number of cases, $\beta_{0}$ is the intercept, *x*_*i*_ is the *i*th measure, and $\varepsilon$ is the random error.

#### GWR

The OLS-based regression model simulates spatial observations by assuming stationary relationships among the variables, but ignores the local variation caused by spatial heterogeneity [35]. However, COVID-19 spread is highly dependent on spatial proximity to infection sources, and thus shows a high degree of spatial autocorrelation [15]. Thus, a GWR was applied to mitigate the influence of spatial autocorrelation. The global Moran’s *I*, the classical measure for spatial autocorrelation [14], was calculated for the confirmed COVID-19 case data, to examine the spatial autocorrelation among COVID-19 cases. The multiscale GWR models were then applied to the COVID-19 case data and space syntax measure data. Finally, global Moran’s *I* was applied to the residuals of the multiscale GWR models to ensure that the residuals were not spatially autocorrelated.

The specialized form of the GWR model for the three explanatory variables is given as follows (Eq. 6), based on the general form defined by Brunsdon et al. [4]:

6 $$y_{i} = \beta_{i0} + \mathop \sum \limits_{k = 1}^{3} \beta_{ik} x_{ik} + \varepsilon_{i}$$

where *i* represents the specific location of each local regression in our study area; $y_{i}$ is the COVID-19 cases at location *i*; $x_{ik}$ is the *k*th explanatory variable at location *i*; $\beta_{i0}$ is the intercept parameter at location *i*; $\beta_{ik}$ is the local regression coefficient for the *k*th explanatory variable at location *i*, which is illustrated as bandwidth in the following sections; and $\varepsilon_{i}$ is the random error at location *i*.

The GWR model performs a location-based calibration of the general linear regression model by putting greater weight on observations nearer to each regression point. The estimation of the local regression coefficient $\beta_{i}$ is performed as follows (Eq. 7):

7 $$\widehat{{\beta_{i} }} = \left( {X^{T} W_{i} X} \right)^{ - 1} X^{T} W_{i} Y$$

where $W_{i}$ is a weight matrix to ensure the observations nearer to *i* are given greater weight. $W_{i}$ is generated by the following Gaussian model-based kernel function [6] (Eq. 8):

8 $$w_{ij} = \exp \left( {\frac{{ - d_{ij}^{2} }}{{\theta^{2} }}} \right)$$

where $w_{ij}$ is the weight value of an observation at location *j* for estimating the coefficient at location *I*, $d_{ij}$ is the Euclidean distance between *i* and *j*, and $\theta$ is a fixed bandwidth size. The fixed bandwidth size is calculated based on the minimum goodness-of-fit measure, the Akaike Information Criterion (AIC). The *corrected AIC* (AICc) is also one of the metrics for evaluating global and local regression models (“Results of GWR with fixed and adaptive bandwidths” section).

#### GWR with adaptive bandwidth

**Fig. 4:**
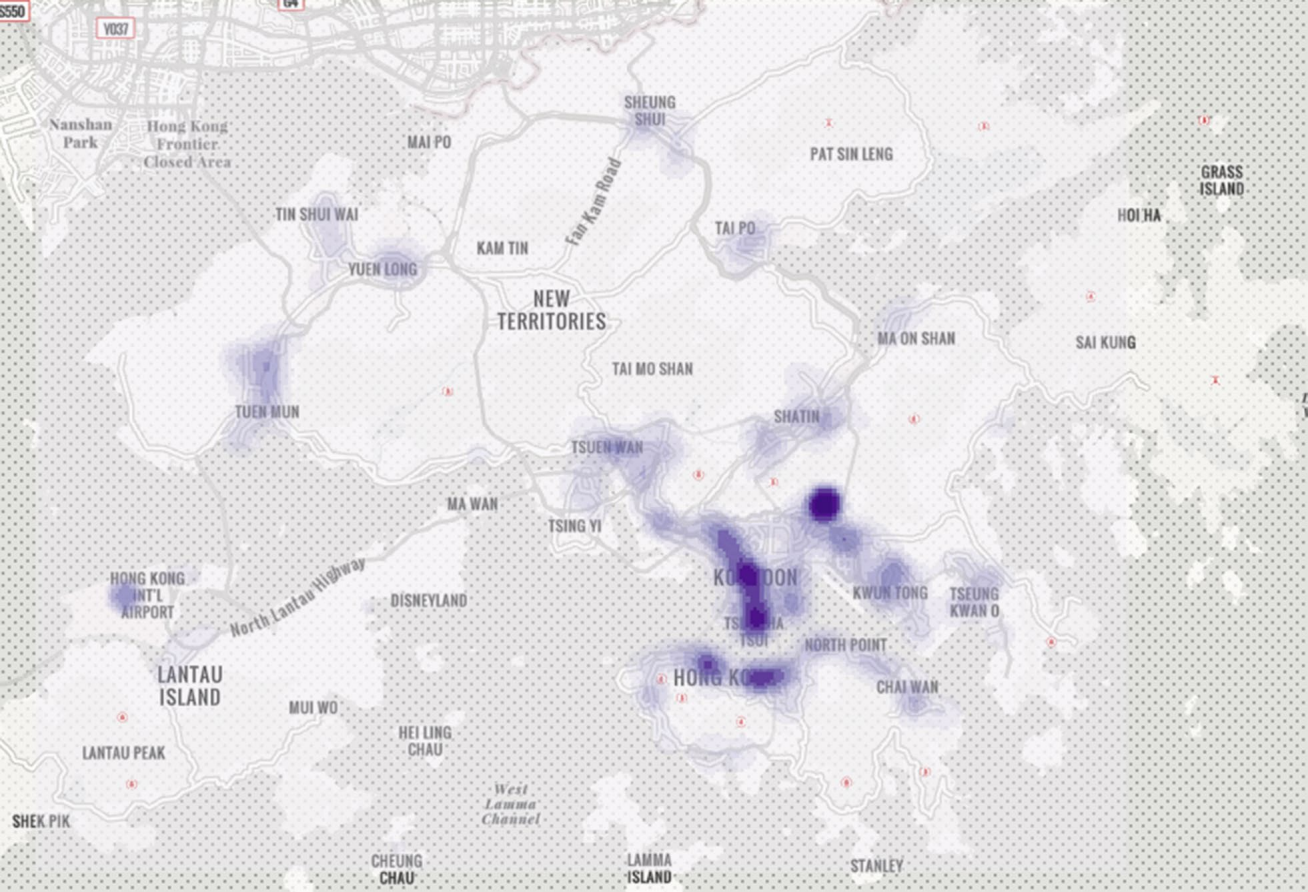
Kernel density of confirmed coronavirus disease 2019 cases in Hong Kong

The GWR model overcomes the assumption of stationarity from global regression. However, the classic GWR model only explains a spatial process on a specific local scale because it assumes a constant relationship, determined by the kernel function and bandwidth, between the dependent and independent variables over space [7, 34]. In practice, it is often difficult to determine the best bandwidth in a spatial regression model. In this study, the epidemic cases and the road network are concentrated in the urban area (Fig. 4). In suburban areas, there are far fewer cases and roads in the surrounding grid cells to participate in the regression, which led to an increase in random error and a decrease in the goodness-of-fit in such an area [27]. In the GWR model with a fixed width, even fewer surrounding grid cells participate in the regression in suburban areas, due to the exclusion of many cells that represented non-residential areas within the fixed bandwidth around the cells of interest. This further reduced the goodness-of-fit in both urban and suburban areas, owing to an overall increase in fitting errors.

Thus, to further calibrate the GWR model, this study used an adaptive bandwidth for the number of neighboring units (i.e., neighboring grid cells) instead of a single geometric distance. That is, the value of $\theta$ in Eq. (8) was further replaced by a series of numbers of nearest neighbors to determine the optimal neighborhood size. The weight and coefficient for each local observation were then calculated according to the adaptive distance between the neighbor and the center. The adaptive bandwidth adjusted the scale of local regression, such that it was smaller in an urban area and larger in a rural area, thereby improving the GWR fitting result and providing a stronger foundation for determining the association between the urban BE and the number of COVID-19 cases.

### Model development and performance measurements

The road network of Hong Kong was selected as the input data for the analysis. Six space syntax measures, one confounding variable, and one essential geometric measure were chosen as candidate independent variables, and the number of COVID-19 cases was set as the dependent variable. The results were then assigned to the grids covering the built area in the study area. We first calculated each measure of space syntax. The measurements represented by depth, degree, and integration are the key factors for predicting the likelihood that human activities occur in a given location. Afterwards, the relationship between the number of confirmed cases in each grid and the average value of each network measure were calculated using univariate variable regression analysis.

Four metrics were used to compare the performances of the regression models, namely *R*^2^, adjusted *R*^2^, AICc, and Moran’s *I* of residuals. *R*^2^ and adjusted *R*^2^ are typical goodness-of-fit measures that represent the proportion of variation in the dependent variable that can be explained by the regression model. AICc is an index for model selection that balances the model complexity and goodness-of-fit, where a lower AICc value indicates better results. The calculation results are then imported into ArcMap to evaluate the spatial autocorrelation of the regression residuals by the Moran’s I. These criteria are used to evaluate whether the GWR models successfully mitigated the spatial heterogeneity or spatial autocorrelation of the data across the study area.

In this study, all the above-mentioned measures of space syntax are calculated from an open source software Place Syntax Tool (version v3.1.4; Meta Berghauser Pont, 2020). The GWR analysis are performed in the software GWR4 (version 4.0.90; Tomoki Nakaya, 2015). The data visualization and layout design are conducted in ArcGIS Pro 2.3 and CorelDRAW X8.

## Experiment results

### Results of univariate regression between individual space syntax measures and COVID-19 cases

**Fig. 5:**
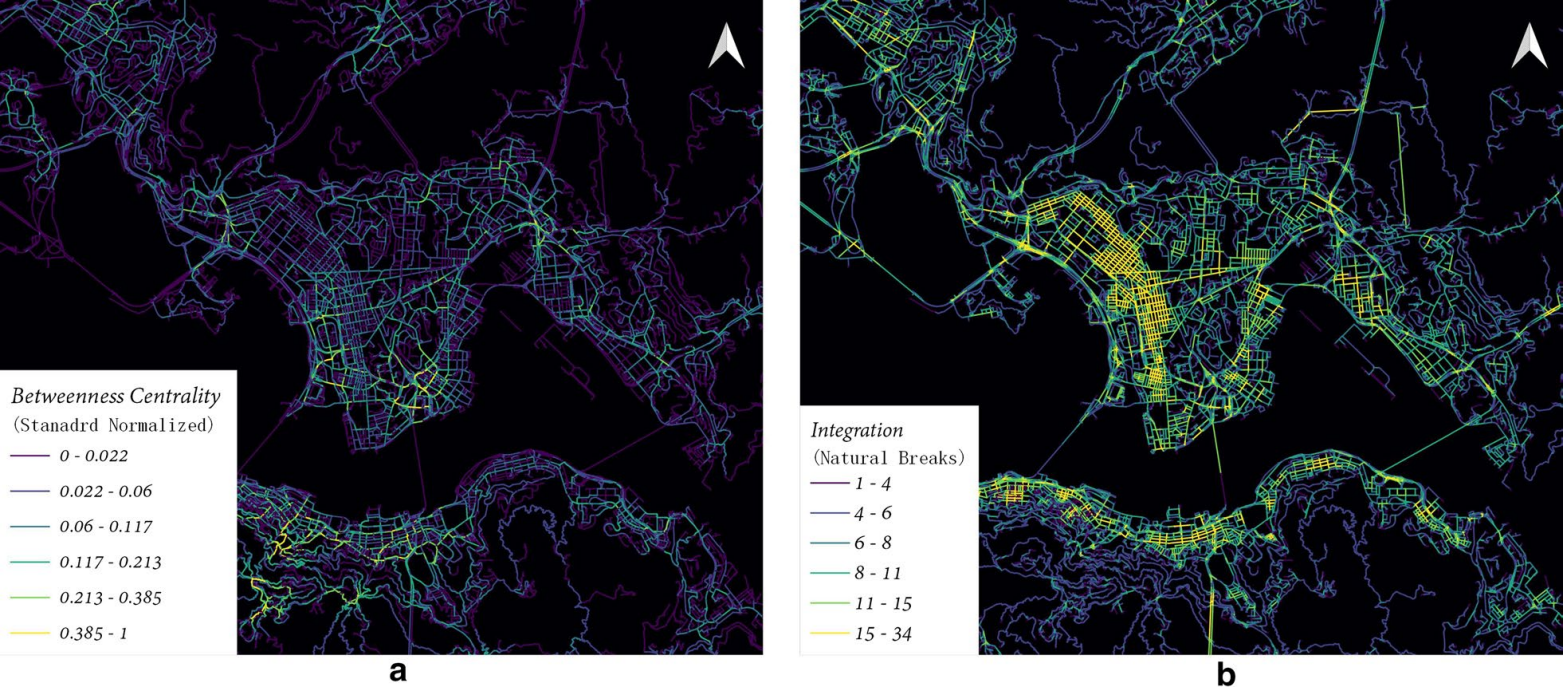
**a** *Betweenness centrality* and **b** *integration* of urban area

The results of the univariate regression between each space syntax measure and COVID-19 incidence are shown in Table 1. Among the space syntax measures, *betweenness centrality* (Fig. 5a) and *integration* (Fig. 5b, and Additional file 1: Figure S1) have the highest *R*^2^ values with respect to the number of confirmed COVID-19 cases. Both measures are positively correlated with the number of confirmed cases, as suggested by the positive *R* values.

**Table 1:** Calculation results of space syntax measures and univariate regression results for predicting the number of cases

Measure	Min	Max	Mean	Stdev.	*R*	Adjusted *R*^2^
Control	0.16	2.0	1.0	0.29	0.30	0.09
Mean depth	1.0	2.73	2.18	0.18	− 0.15	0.02
Global integration	0.79	3.49	1.35	0.45	0.42	0.17
Total depth	4.0	219.0	34.7	24.29	− 0.26	0.07
Degree	1.0	15.0	3.22	1.41	0.38	0.14
Betweenness centrality	0	37,920.0	1341.67	1509.26	0.59	0.34
Length	12.81	2687.72	76.59	69.78	0.32	0.10
Population density	–	–	–	–	0.44	0.20

*Control* (Additional file 1: Figure S2), *degree* (Additional file 1: Figure S3), and *betweenness centrality* are all indicators of the importance of a certain area in an entire space. The first two measures only consider the topological characteristics of a network, whereas *betweenness centrality* considers both the topological features and the number of shortest paths passing the network location of concern. Therefore, *betweenness centrality* showed the best performance in predicting the number of cases. A higher *depth* value means that a location is less likely to be a place in which human activities occur. Thus, as expected, *depth* (Additional file 1: Figure S4) is negatively correlated with the number of confirmed cases, but the *R*^2^ value is low, which shows that depth is not a good indicator for explaining the number of confirmed cases. Compared with the density of human activities, as reflected by depth, the chance of encountering and having social contact with people from other areas as reflected by betweenness centrality is more related to the risk of developing COVID-19.

The overall results of these single linear regressions indicate that places with higher accessibility tend to have more confirmed cases of COVID-19 (Figs. 4 and 5). This is probably because areas with a higher risk of exposure lead to a higher risk of infection, but more evidence needs to be obtained to confirm this.

### Results of OLS-based linear regression model

Before the multiple regression was conducted, the multicollinearity of the independent variables was tested by calculating the least variance inflation factor (VIF). The three variables with a VIF of less than five [33], namely *integration*, *betweenness centrality*, and *length*, representing the topology, the nearest distance, and the geometric length of the street network, respectively, were used in the multiple regression*.* Previous studies have suggested that the integration of network topology and network length affords a better estimation of the relationship between human activity and the urban road network [26, 43], which validates the effectiveness of the three selected variables to some extent.

Table 2 summarizes the results of the OLS analysis. All three variables are positively associated with the COVID-19 incidences, which is consistent with the univariate regression results. The *R*^2^ and adjusted *R*^2^ of the OLS model, while markedly improved compared with those of the univariant regression, are slightly greater than 0.4. The number of COVID-19 cases across the study area has a global Moran’s *I* equal to 0.2747, with a *z*-score of 33.38 and *p*-value of < 0.001, thereby indicating that the distribution of COVID-19 cases is highly spatially autocorrelated and clustered. Thus, the relatively low fitness for the OLS model may be mainly due to the inability of OLS to capture local variations in the highly spatially autocorrelated distribution of COVID-19 cases.

**Table 2:** Ordinary least-squares regression of coronavirus disease 2019 cases

Measure	Coefficient	*t*-statistic	*p*-value	VIF^a^
(*R*^2^: 0.4220; Adjusted *R*^2^: 0.4199)				
Integration	0.2342	3.6570	0.0004	3.4278
Betweenness	0.2637	2.9701	0.0000	2.9311
Length	− 0.1029	− 1.1027	0.0000	2.0325
Population density	0.0113	6.8133	0.0002	1.3697

^a^VIF refers to variance inflation factor

### Results of GWR with fixed and adaptive bandwidths

The results of the GWR using between space syntax measures and COVID-19 cases with fixed and adaptive bandwidths are shown in Table 3. The fixed bandwidth of the GWR is 10,900 m, which is the value that minimizes the AICc of the regression result. However, the fixed bandwidth is relatively large when apply to local areas for spatial regression [2]. In GWR analysis, a larger bandwidth does not allow for much local fitting within each moving window and the model also approached the global model. Thus, three adaptive bandwidths of the GWR with 100, 150, and 200 neighbors were used to calibrate the spatial variation across the entire study area (Table 3). These bandwidths allow the model to fit spatial differences at a smaller local scale. There are a total of 1112 grids in the study area.

**Table 3:** Measures of goodness-of-fit for geographically weighted regression (GWR) models (fixed and adaptive models)

Criterion	OLS	GWR			
Model	–	(a)	(b)	(c)	(d)
Bandwidth	–	Fixed (10,205 m^a^)	100 neighbors	150 neighbors	200 neighbors
*R*^2^	0.441	0.493	0.588	0.555	0.536
Adjusted *R*^2^	0.439	0.477	0.523	0.511	0.501
AICc^b^	8195	8129	8103	8097	8101
*z*-score of residuals^c^	5.877	2.316	1.933	− 1.285	− 0.255

^a^The automatically selected value that minimizes the Akaike information criterion (AICc)

^b^AICc refers to the corrected Akaike information criterion

^c^*z*-score of residuals (significantly dispersed: < − 1.96; random: − 1.96 to 1.96; significantly clustered: > 1.96)

Compared with those of the OLS-based regression, the GWR with a fixed bandwidth achieves a higher *R*^2^/adjusted *R*^2^ and a lower AICc (Table 3), which demonstrates that GWR better captures the spatial dependency of the distribution of COVID-19 cases than OLS. However, the residuals of the regression are still significantly clustered according by *z*-score, thereby showing that a portion of the spatial dependency was is not captured by the model.

As can be seen, GWR models with adaptive bandwidth (Models b, c, and d) achieve better adjusted *R*^2^ and AICc values than the fixed-bandwidth GWR model. Furthermore, the *z*-scores of the residuals of these former models fall in a range that indicates spatial randomness, thereby indicating that most of the spatial dependency within the data was captured by the regression. The best performance, i.e., the highest *R*^2^ and adjusted *R*^2^ and the lowest AICc values, was achieved by Model b with 100 neighbors. The results demonstrate that the GWR models with an adaptive bandwidth can better capture the local variations in COVID-19 cases than the GWR with a fixed bandwidth. The variation in the scales of spatial dependency at different locations, in terms of absolute distances, are better represented and captured by the adaptive bandwidth. This enables the GWR models with an adaptive bandwidth to model the relationships between the urban BE and COVID-19 cases more reliably.

**Fig. 6:**
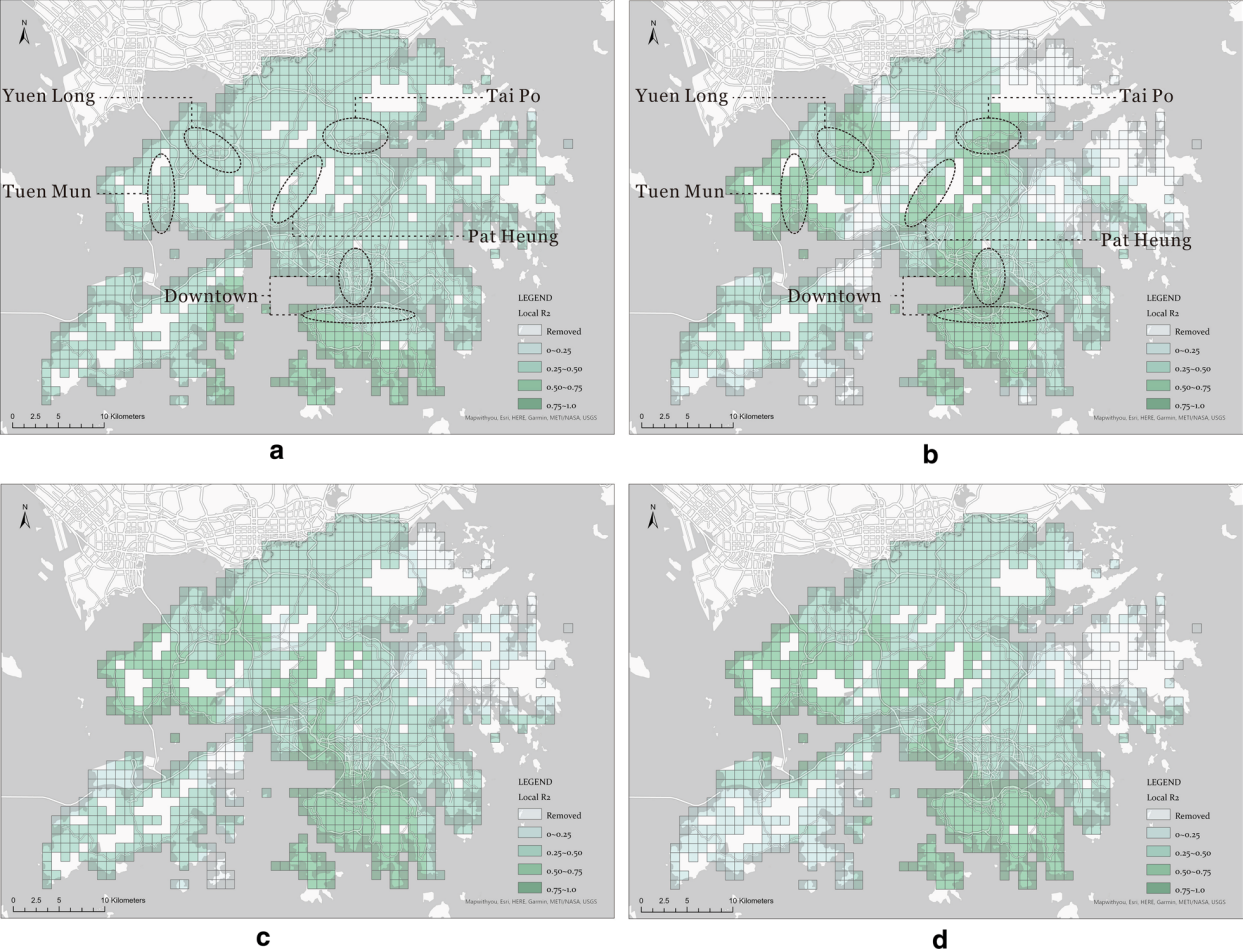
Distribution of the local *R*^2^ values of the geographically weighted regression models with **a** a fixed bandwidth; **b** bandwidth = 100 neighbors; **c** bandwidth = 150 neighbors; and (d) bandwidth = 200 neighbors. “Removed” indicates the removed non-residential areas that do not participate in the regression

The GWR models with an adaptive bandwidth not only obtained better overall fitting results, as shown in Table 3, but also obtained higher goodness-of-fit in local areas. In the downtown area, in which the number of COVID-19 confirmed cases is highest (Fig. 6), the GWR Models (b), (c), and (d), which have an adaptive bandwidth, have higher local *R*^2^ values than those of Model (a), which has a fixed bandwidth. Areas with local *R*^2^ values higher than 0.75 (grids in dark green in Fig. 6) cover most of the urban areas, such as 48% and 42% of all of the grid cells for Model (c) and Model (d). Model (a) results in local *R*^2^ values higher than 0.75 in only 23% of the grid cells. Models (b), (c), and (d) also have higher local *R*^2^ values than Model (a) in Tuen Mun, Yuen Long, and Tai Po, which are the three “satellite towns” where confirmed cases are clustered (Fig. 6a, b). The fitting results of Models (c) and (d) are not as good as those of Model (b) because the models gradually approach global regression when the bandwidths increase.

To further explain the improvement of the adaptive bandwidth method to the degree of fit on the local scale (Table 4), three representative regions were selected for comparison. Kowloon Peninsula and North Hong Kong Island (downtown area in Fig. 6a, b) are selected to represent the metropolitan area, Yuen Long Town represents the new town area, and Pat Heung represents the rural area. The division of urban and rural areas is based on the official land use document from the Hong Kong Planning Department [38].

**Table 4:** Local R^2^ for downtown area, satellite town area and rural area

Measure	R^2^	Mean local R^2^		
		Downtown	Satellite town	Rural areas
Model (a)	0.493	0.460	0.406	0.417
Model (b)	0.588	0.561	0.501	0.526
Model (c)	0.555	0.521	0.467	0.488
Model (d)	0.536	0.494	0.473	0.350

## Discussion and conclusion

Human cognition of a space is based on not the entirety of a city, but its small subdivided parts [16, 30]. It is thus essential to decompose a large urban space into smaller and unique spatial units to perform quantitative analysis of the differences between city areas. The onset risk prediction, spatial distribution, and pattern of spread of COVID-19 have been recognized in previous research. However, most studies emphasized on the physical built environment on the relatively coarse scale, such as road and building density at each county or city. The lack of discussion of the relationship between COVID-19 and spatial configuration of urban environment especially the road network prevents us from perceiving how COVID-19 interacts with this key factor.

This study found that there is a clear and strong correlation between COVID-19 incidence and space syntax measures after controlling the possible influencing factors. A group of six space syntax measures representing the urban BE and one confounding factor population density were applied to explain the geographic distribution pattern of COVID-19 cases in Hong Kong. The univariate analysis suggests that COVID-19 incidence is highly dependent on integration and betweenness measures and less dependent on degree and length. Interestingly, the population density shows a relatively weak correlation compared with other measures, which is contrast to most public health research that the population density is always regarded as a critical factor associated with the risk of infectious [46]. However, this finding embraces some similar evidence in the Hong Kong context. For instance, KAN [18] found that population density had no significant relationship with locations visited when investigating the covid-19 spread in the Hong Kong context. Also, Lai et al. [23] have investigated the diffusion patterns of the severe acute respiratory syndrome (SARS) during 2003 in Hong Kong and found that people in urban areas had a higher risk of contracting the disease than those in rural areas irrespective of population density. Still, the distribution of COVID-19 cases shows quite different trends.

Besides, this study proves the effectiveness of a GWR model with adaptive bandwidth in predicting the diffusion of covid-19 cases. This study utilizes a GWR model with adaptive bandwidths that considers spatial heterogeneity across the urban and rural areas. As the distribution of COVID-19 cases is heterogeneous in urban and rural areas, the bandwidth of the GWR model must be adjusted accordingly. This calibration greatly improves the GWR fitting results and aids determination of the association between the urban BE and the distribution of COVID-19 cases. Specifically, we adjusted the bandwidth to be smaller in urban areas and larger in rural areas, based on the pattern of distribution of COVID-19 cases. Overall, the goodness-of-fit of this model outweighs that of the OLS model and the normal GWR model with a fixed bandwidth. As most previous studies on the spread of infectious diseases have claimed that high-dense urban areas are more conducive to the spread of infectious diseases like flu and H1N1, urban centers have a higher risk of onset than rural areas. However, this study highlights an important but neglected factor: the spatial configuration of the street network, which would partly explain how the infection spreads and show a high degree of consistency in urban, new town, and rural areas in Hong Kong. That is, a more integrated street may increase the chance of infections. This also echoes the research of the view that rural areas and suburban sprawl are not necessarily safer spaces during the COVID-19 crisis [5]. Analyzing the spatial configuration of urban built environment may be a new dimension to explain the characteristics of urban and rural epidemics in the future.

Finally, this study also suggests that infectious diseases, such as COVID-19, should be explored on a more local scale. The topology, network accessibility, and centrality of an urban area were proven to be effective for use in predicting the spread of COVID-19. The GWR model with bandwidth of 100 neighbors best explains the COVID-19 incidence in Hong Kong. This indicates that the correlation between the geographic distribution of COVID-19 cases and the urban BE may be more relevant on a local scale than on a global scale within a single city. Quantitative analysis combined with travel radius in the future should lead to more accurate conclusions. As the world is currently experiencing a winter rebound of COVID-19 outbreaks, this study may provide a key reference and stimulate further studies to understand the relationship between the urban BE and health, during and beyond the COVID-19 pandemic. This research aims to serve as a reference for understanding the geographical distribution pattern of COVID-19 cases and inspire new approaches in density management that will help long-term survival in future pandemics.

However, there are still some limitation exists in this study. The first is data uncertainty. Although the cases data is the highest quality and most detailed that can be collected in Hong Kong, the geographical location information of the cases data used in this study are extracted from the location of onset or residence declared by each case. This location may not be completely accurate, and probably not the places they contracted the COVID-19 virus. The second limitation is the selection of the explanatory variables. This research focuses on the spatial distribution pattern of COVID-19 cases from a purely spatial structure perspective and only selects variables representing spatial characteristics for establishing the spatial regression models. Actually, the urban space structure is only one of the main factors that affect the spread of infectious diseases. Other factors such as population density, socioeconomic background, and the availability of medical facilities may worthwhile for further investigation to predict the distribution of COVID-19 in more detail and quantitative level.

## Supplementary Information

**Additional file 1: Figure S1.** Integration. **Figure S2.** Control. **Figure S3.** Degree. **Figure S4.** Total depth.

## Data Availability

All data and materials used are public accessed as illustrated in the article.
