# Supplementary figures and images for: Examining the diffusion of coronavirus disease 2019 cases in a metropolis: a space syntax approach
Source: Int J Health Geogr. 2021 Apr 29;20:17. doi: 10.1186/s12942-021-00270-4 (PMC8083925; doi:10.1186/s12942-021-00270-4)

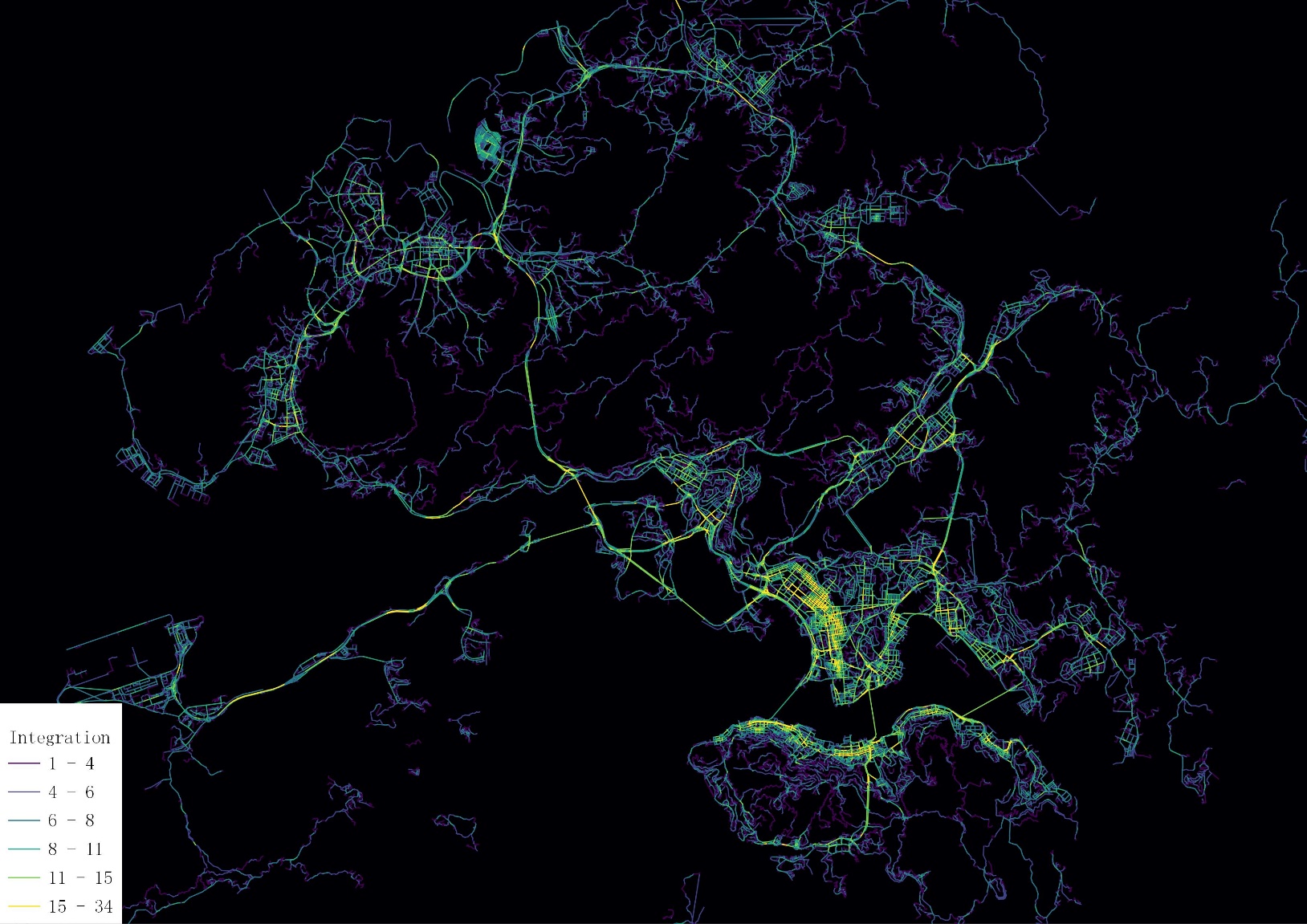


Figure S1 Integration


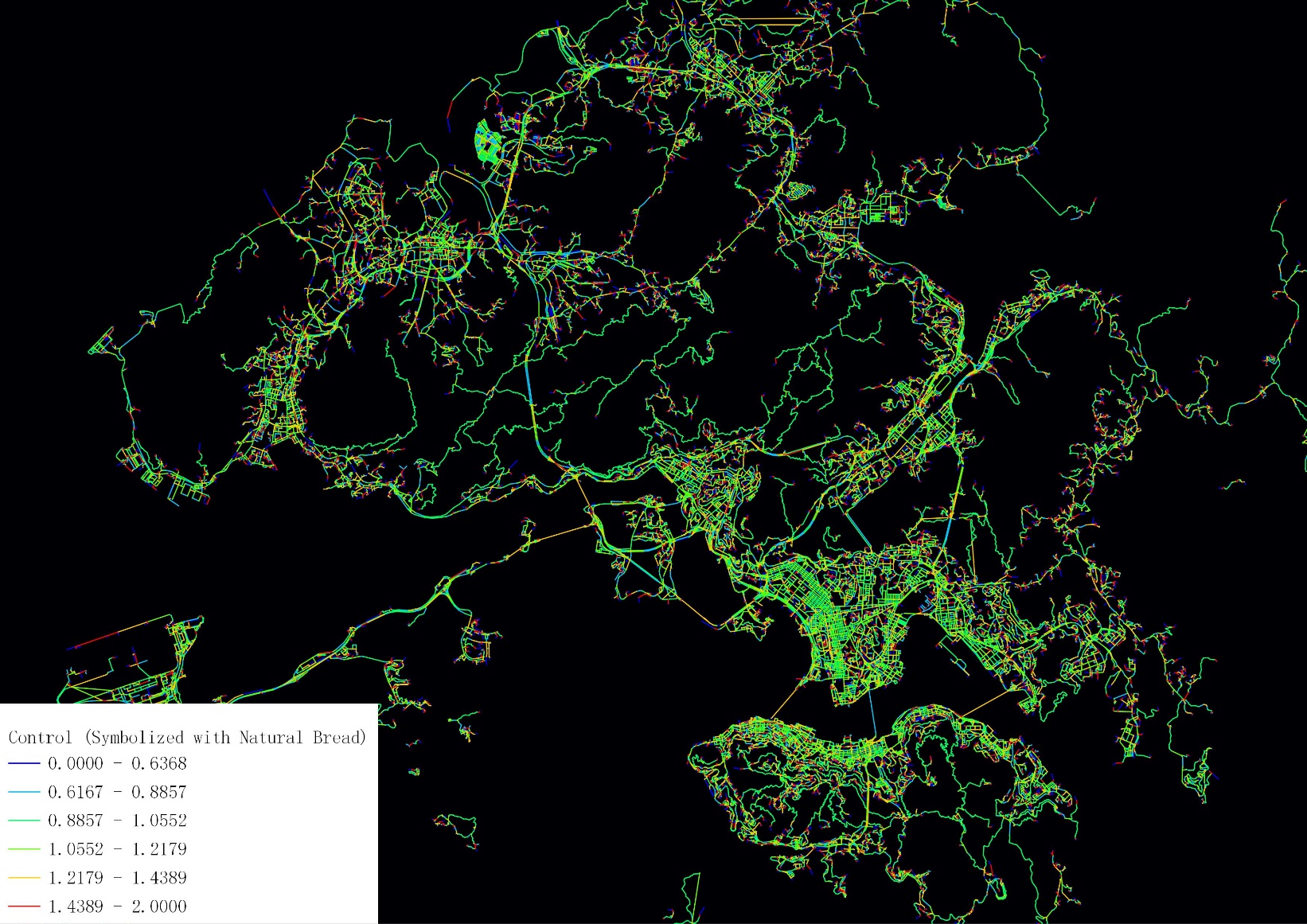


Figure S2 Control


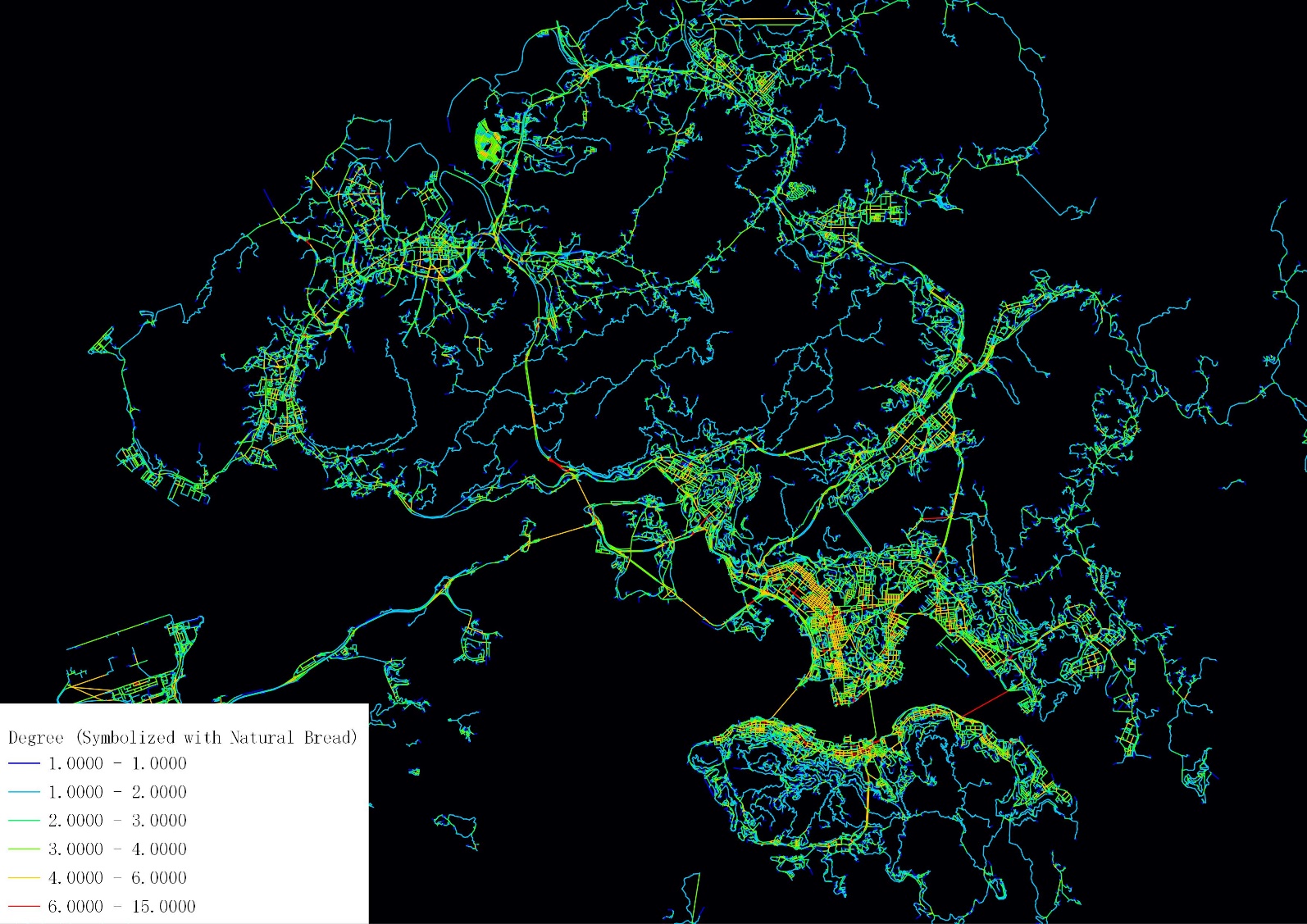


Figure S3 Degree


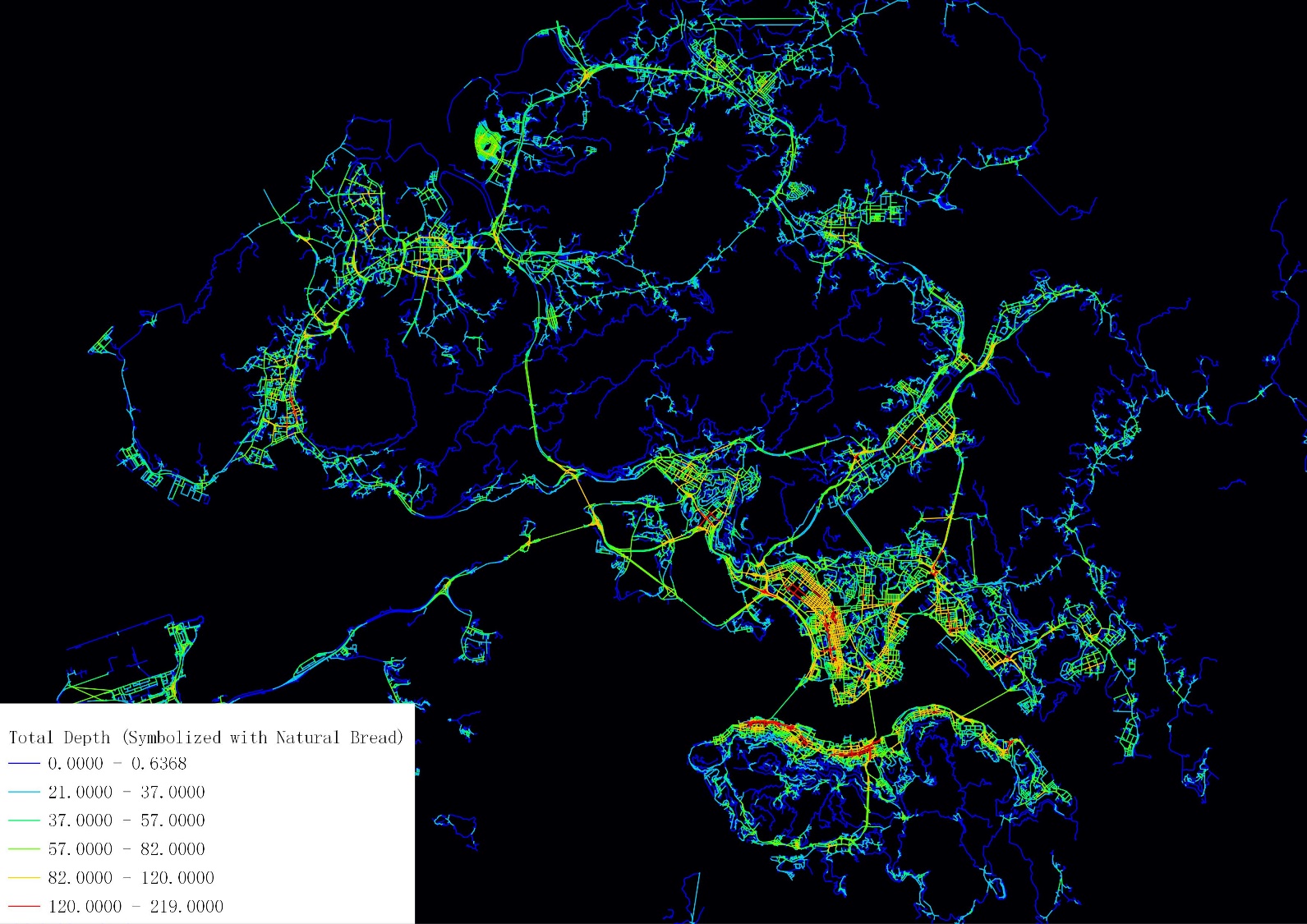


Figure S4 Total Depth

Supplement: Supplementary file 1 — Additional file 1: Figure S1. Integration. Figure S2. Control. Figure S3. Degree. Figure S4. Total depth. [file 12942_2021_270_MOESM1_ESM.docx]
